# On the topological surface states of the intrinsic magnetic topological insulator Mn-Bi-Te family

**DOI:** 10.1093/nsr/nwad066

**Published:** 2023-03-09

**Authors:** Yuan Wang, Xiao-Ming Ma, Zhanyang Hao, Yongqing Cai, Hongtao Rong, Fayuan Zhang, Weizhao Chen, Chengcheng Zhang, Junhao Lin, Yue Zhao, Chang Liu, Qihang Liu, Chaoyu Chen

**Affiliations:** Shenzhen Institute for Quantum Science and Engineering (SIQSE) and Department of Physics, Southern University of Science and Technology (SUSTech), Shenzhen 518055, China; Shenzhen Institute for Quantum Science and Engineering (SIQSE) and Department of Physics, Southern University of Science and Technology (SUSTech), Shenzhen 518055, China; Shenzhen Institute for Quantum Science and Engineering (SIQSE) and Department of Physics, Southern University of Science and Technology (SUSTech), Shenzhen 518055, China; Shenzhen Institute for Quantum Science and Engineering (SIQSE) and Department of Physics, Southern University of Science and Technology (SUSTech), Shenzhen 518055, China; Shenzhen Institute for Quantum Science and Engineering (SIQSE) and Department of Physics, Southern University of Science and Technology (SUSTech), Shenzhen 518055, China; Shenzhen Institute for Quantum Science and Engineering (SIQSE) and Department of Physics, Southern University of Science and Technology (SUSTech), Shenzhen 518055, China; Shenzhen Institute for Quantum Science and Engineering (SIQSE) and Department of Physics, Southern University of Science and Technology (SUSTech), Shenzhen 518055, China; Shenzhen Institute for Quantum Science and Engineering (SIQSE) and Department of Physics, Southern University of Science and Technology (SUSTech), Shenzhen 518055, China; Shenzhen Institute for Quantum Science and Engineering (SIQSE) and Department of Physics, Southern University of Science and Technology (SUSTech), Shenzhen 518055, China; Shenzhen Institute for Quantum Science and Engineering (SIQSE) and Department of Physics, Southern University of Science and Technology (SUSTech), Shenzhen 518055, China; Shenzhen Institute for Quantum Science and Engineering (SIQSE) and Department of Physics, Southern University of Science and Technology (SUSTech), Shenzhen 518055, China; Shenzhen Institute for Quantum Science and Engineering (SIQSE) and Department of Physics, Southern University of Science and Technology (SUSTech), Shenzhen 518055, China; Shenzhen Institute for Quantum Science and Engineering (SIQSE) and Department of Physics, Southern University of Science and Technology (SUSTech), Shenzhen 518055, China

**Keywords:** intrinsic magnetic topological insulator, topological surface states, magnetic gap, magnetic reconfiguration, topological surface state redistribution, van der Waals spacing expansion

## Abstract

We review recent progress in the electronic structure study of intrinsic magnetic topological insulators (MnBi_2_Te_4_) · (Bi_2_Te_3_)_n_ ($n\ = \ 0,\ 1,\ 2,\ 3$) family. Specifically, we focus on the ubiquitously (nearly) gapless behavior of the topological Dirac surface state observed by photoemission spectroscopy, even though a large Dirac gap is expected because of surface ferromagnetic order. The dichotomy between experiment and theory concerning this gap behavior is perhaps the most critical and puzzling question in this frontier. We discuss various proposals accounting for the lack of magnetic effect on the topological Dirac surface state, which are mainly categorized into two pictures, magnetic reconfiguration and topological surface state redistribution. Band engineering towards opening a magnetic gap of topological surface states provides great opportunities to realize quantized topological transport and axion electrodynamics at higher temperatures.

## Teaser

This work reviews recent progress in the electronic structure study of intrinsic magnetic topological insulators Mn-Bi-Te family, discusses the puzzling behavior of the topological surface state subject to bulk magnetic order, and potential route to realize the magnetic gap towards high temperature topological quantized transport.

## INTRODUCTION

**Figure 1.**
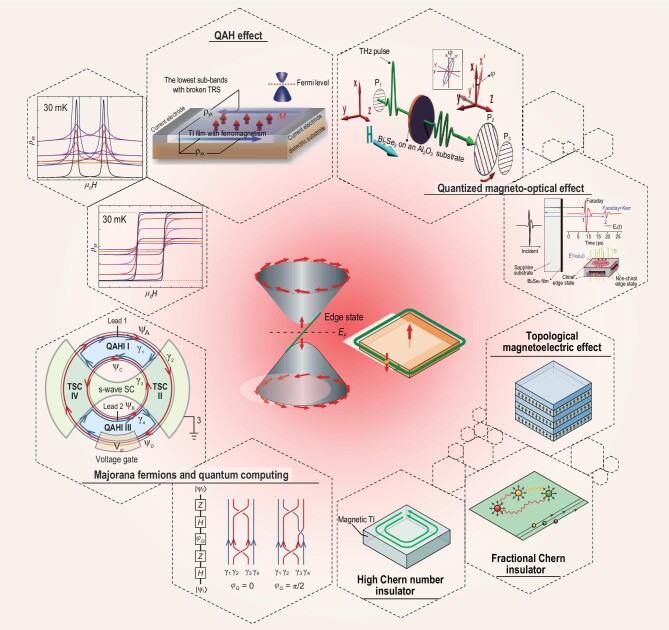
Emergent topological phenomena arising from an intrinsic magnetic TI. The interplay of magnetism and band topology offers a great chance to explore the QAHE previously realized on magnetically doped TI films (adapted from [24]) but now potentially at much higher temperatures, the chiral Majorana fermion at the interface of QAHE state and s-wave superconductor which forms the basis of topological quantum computing (from [35]), quantized magneto-optical effect (from [36]), topological magnetoelectric effect, fractional Chern insulator, high Chern number insulator and so on (from [37]).

Magnetism has been used and studied over millennia. Yet, this branch of physics keeps flourishing in recent years with emerging states of magnetic matters such as quantum spin liquid [1,2] and two-dimensional (2D) magnets [3,4]. By comparison, the first two decades of the new millennium embraced the triumph of topological states of matter, which has revolutionized our knowledge of crystalline materials by introducing topological invariants to categorize their electronic structure [5,6]. In 2007, the realization of the quantum spin Hall effect (QSHE) based on a 2D HgTe/CdTe quantum well [7,8] opened a new era of exploring topological phases and materials in condensed matter. Electronically, this QSH state is insulating with a bulk gap separating the conduction and valence bands but has a pair of one-dimensional (1D) conducting edge states. These 1D topological edge states are protected by time-reversal symmetry, wherein elastic backscattering by nonmagnetic impurities are forbidden, holding the potential for dissipationless spintronics. The QSHE state can be classified by a type of topological invariant called the ${Z}_2$ invariant [9] and is now recognized as the first example of a 2D time-reversal invariant topological insulator (TI) with ${Z}_2 = 1$. ${Z}_2$ classification can be generalized to three-dimensional (3D) to describe ‘weak’ and ‘strong’ TIs [10]. A 3D strong TI has a ubiquitous, gapless topological surface state (TSS) with helical spin texture due to spin-momentum locking. In 2008, 3D TI was first realized based on Bi-Sb alloys [11] with multiple TSSs crossing the Fermi level five times. Up to now, there have been hundreds of materials predicted as 3D strong TI [12–14] and dozens of them have been experimentally verified, usually through direct observation of their Dirac cones at the surface by angle-resolved photoemission spectroscopy (ARPES) [15–17]. Among them, the most representative one is Bi_2_Se_3_ and its family of materials [18–21] found in 2009. The Bi_2_Se_3_ family is now considered the ‘hydrogen atom’ of topological materials due to its simple and elegant electronic structure. It is a semiconductor with a bulk gap of $\sim 0.3\ {\rm eV}$, among the few with sizable bulk gap to manifest surface state transport. It has only one TSS Dirac cone at the center of the Brillouin zone, with the Dirac point located close to the middle of bulk gap. It consists of -Se-Bi-Se-Bi-Se- quintuple layers (QLs) stacking along *c* axis bounded by van der Waals (vdW) interaction, easy for device fabrication and epitaxial growth. Importantly, the TSS is confirmed to be robust in ambient environments because of the topological protection [22]. These excellent properties distinguish the Bi_2_Se_3_ family from others for the exploration of novel topological effects. Particularly, the quantum anomalous Hall effect (QAHE), a time-reversal-symmetry-breaking version of QSHE, was predicted in 2010 [23] and then realized in 2013 [24] based on magnetically doped films of this family (Fig. 1).

Our story of topological states of matter has reached a point where this rising star meets a classic field of physics, magnetism. However, this is not their first rendezvous. Back in 1980, the quantum Hall effect (QHE) was observed from a 2D electron gas system subjected to a strong magnetic field [25]. This phenomenon was later explained theoretically as a topological property of the occupied bands in the Brillouin zone [26]. In the current context, we can call the QHE the first topological insulator ever found, classified by a time-reversal-symmetry-breaking topological invariant called Chern number *C* (originally the well-known Thouless–Kohmoto–Nightingale–Nijs invariant [26]). The applied magnetic field in QHE can be replaced by the intrinsic magnetization of the material, leading to QAHE. In this regard, QHE and QAHE states are both Chern insulators. In 2D Chern insulators, 1D gapless edge states emerge at the boundary between the Chern insulator and ordinary insulator (vacuum) because of the distinct band topology. Like the 1D helical edge states of QSHE, the 1D chiral edge state in QAHE can propagate along one direction with forbidden backscattering, suitable for developing low-power-consumption electronics without the need for an applied magnetic field.

The prediction and realization of QAHE [23,24] represent a breakthrough in fundamental physics, yet much effort is needed toward its practical application. On the one hand, Hall bar devices based on magnetically doped films require sophisticated epitaxial growth and microfabrication procedures that only a few labs in the world can accomplish [24,27–34]. On the other hand, atomic doping brings disorder to the crystal and inhomogeneity to the electronic structure, resulting in a much reduced effective exchange gap of the TSS, where the realization temperature was as low as $30\ {\rm mK}$ at first [24] and the current record is $2\, {\rm K}$ [30] after 10 years’ effort. In this context, intrinsic magnetic TI, which combines magnetic order and band topology in the same material without the need of doping, is highly desired.

In a magnetic TI, while the time-reversal symmetry $\Theta $ is broken by the magnetic order, its combination with certain magnetic lattice symmetry such as rotation ${C}_n$ and fractional translation ${T}_{1/2}$ can retain an equivalent time-reversal symmetry and the system can still be classified by the topological ${Z}_2$ invariant. This was first discussed in the theoretical proposal of antiferromagnetic TI in 2010 [38]. In an antiferromagnetic TI, both $\Theta $ and ${T}_{1/2}$ are broken but the combination $S\ = \ \Theta {T}_{1/2}$ is preserved, leading to a topologically nontrivial phase which shares the topological ${Z}_2$ invariant and quantized magnetoelectric effect in a 3D strong TI. The difference is that, while 3D TIs have symmetry-protected gapless TSS at all surfaces, 3D antiferromagnetic TIs have intrinsically gapped TSS at certain surfaces with broken *S* symmetry. The gapped TSSs in 3D antiferromagnetic TIs carry a half-quantized Hall conductivity (${\sigma }_{xy} = {e}^2/2h$), which may aid experimental confirmation of quantized magnetoelectric coupling (Fig. 1). Although with fascinating properties, the material realization of an intrinsic antiferromagnetic TI was not initiated until 2017. First-principles calculation proposed that by inserting an MnTe bilayer into the first quintuple layer of Bi_2_Te_3_, a septuple layer of MnBi_2_Te_4_ is constructed and the TSS opens a sizable magnetic gap, promising a robust QAH state [39,40]. The material was first experimentally realized with thin films via molecular beam epitaxy [41]. Theoretical works on single crystalline MnBi_2_Te_4_ were reported in 2019 and revealed its fertile topological states of matter [42–45]. Since the successful preparation of single crystal MnBi_2_Te_4_, the surge of intrinsic magnetic TI based on MnBi_2_Te_4_ and its family of materials has begun.

Now we know more details concerning the structural, magnetic, and topological aspects of MnBi_2_Te_4_ antiferromagnetic TI. Its $R\bar{3}m$ lattice consists of layered -Te-Bi-Te-Mn-Te-Bi-Te- units (SL) stacking along the *c* axis and the layers are bounded by vdW force [46,47]. In the ground state below ${T}_N\sim 24.6\, {\rm K}$, Mn ions ($S = 5/2$ of $2 + $ valence) with a large magnetic moment of $\sim 5\ {\mu }_B$ form a ferromagnetic layer with moments pointing out-of-plane. These ferromagnetic layers couple each other in an antiferromagnetic way along the *c*-axis [48,49] (A-type AFM). The bulk gap is around $220\ {\rm meV}$ from calculation [45] but only $\sim 130\ {\rm meV}$ as directly observed by ARPES [50–52]. At the natural cleavage plane (0001), the TSS gap of $\sim 88\ {\rm meV}$ at the Dirac point is expected due to *S* breaking [42–45]. This sizable TSS gap is the key ingredient enabling the observation of exotic phenomena such as quantized magnetoelectric coupling [38,53,54], axion electrodynamics [55–57], QAHE [24,27–34,58], and chiral Majorana fermions [59–62] at much higher temperatures. In fact, quantum transport experiments have revealed the existence of a 2D Chern insulator with QAHE observed at $1.4\ {\rm K}$ based on 5 SLs [63], and the characteristics of an axion insulator state based on 6 SLs [64], both at zero magnetic field. Under a perpendicular magnetic field ($15\ {\rm T}$), characteristics of high-Chern-number quantum Hall effect without Landau levels contributed by dissipationless chiral edge states are observed, indicating a well-defined Chern insulator state with $C\ = \ 2$ (9, 10 SLs) [65]. Its intralayer ferromagnetic and interlayer antiferromagnetic configuration exhibits the layer Hall effect in which electrons from the top and bottom layers deflect in opposite directions due to the layer-locked Berry curvature, resulting in the characteristic of the axion insulator state (6 SLs) [66]. In addition to these experimental observations, it is further shown by theory that manipulating its magnetic and structural configuration can give rise to many new topological states. For example, the flat Chern band in twisted bilayer MnBi_2_Te_4_ may boost the fractional Chern insulator and $p + ip$ topological superconductor [67]; changing the stacking order between MnBi_2_Te_4_ SL and Bi_2_Te_3_ QL may lead to novel states such as QSHE insulator with and without time-reversal symmetry [68]; magnetic ground states other than A-type AFM may lead to different phases such as Weyl semimetal [69,70] and higher-order topological Möbius insulator [71]. These predictions (Fig. 1) certainly deserve further experimental efforts.

There have been several reviews/perspectives on this intrinsic magnetic TI family [72–76], with distinct emphases on theoretical, computational, and transport studies, respectively. In this review, we focus on the electronic structure of (MnBi_2_Te_4_) · (Bi_2_Te_3_)_n_ ($n\ = \ 0,\ 1,\ 2,\ 3$) family, which has long been a subject of much debate. We will first review the ARPES observation of ubiquitously (nearly) gapless behavior of TSS Dirac cone from MnBi_2_Te_4_ and MnBi_2_Te_4_ SL termination, as well as the band hybridization features from Bi_2_Te_3_ QL terminations. While the significantly reduced TSS gap size of MnBi_2_Te_4_ termination deviates from that obtained by first-principles calculations, it is not against the relatively low temperature for the observation of QAHE, suggesting that the effective magnetic moments for the TSS may be diminished. There is also experimental evidence suggesting the robust A-type AFM order at the topmost SL layers from magnetic force microscopy (MFM), polar reflective magnetic circular dichroism (RMCD) and X-ray magnetic circular/linear dichroism (XMCD/XMLD) measurements. Furthermore, the magnetic splitting of certain bulk quasi-2D bands seems to validate the effect of magnetic order on the low-energy band structure. Bearing these established experimental results in mind, we then discuss the validity of possible scenarios proposed to account for the (nearly) gapless TSS from MnBi_2_Te_4_ termination, such as surface magnetic reconstruction, TSS redistribution, defect and self-doping effects, etc. Future band engineering towards opening a magnetic gap at the TSS Dirac point via approaches such as magnetic manipulation, element substitution, chemical potential and material optimization is proposed, which would provide great opportunities to the realization of QAHE and topological magnetoelectric effect at higher temperatures.

## UBIQUITOUSLY GAPLESS TSSs

**Figure 2.**
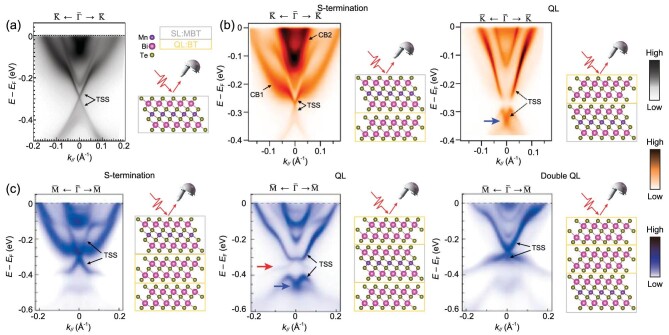
Gapless TSSs from all the terminations of (MnBi_2_Te_4_) · (Bi_2_Te_3_)_n_ ($n\ = \ 0,\ 1,\ 2$). (a) Gapless TSS from the (0001) surface of MnBi_2_Te_4_, measured at $10\ {\rm K}$ using photon energy $hv\ = \ 6.3\ {\rm eV}$ [50]. (b) Gapless TSSs from the SL (left) and QL (right) terminations of MnBi_4_Te_7_, measured at $11\ {\rm K}$ using photon energy $hv\ = \ 6.3\ {\rm eV}$ [85]. (c) Gapless TSSs from the SL (left), QL (middle) and double QL (right) terminations of MnBi_6_Te_10_, measured at $6\ {\rm K}$ using photon energy $hv\ = \ 6.994\ {\rm eV}$ [79]. Red arrow emphasizes the in-gap states inside the hybridization gap between TSS and bulk valence band while blue arrow indicates the TSS Dirac point.

Due to the *S* breaking at the natural cleavage plane (0001), below ${T}_N\sim 24.6\ {\rm K}$ antiferromagnetic TI MnBi_2_Te_4_ is expected to show a magnetic gap $\sim 88\ {\rm meV}$ at the Dirac point of TSS [42–45]. Earlier ARPES investigations on the single crystals reported gapped TSS behavior with a Dirac gap ranging from $70\ {\rm meV}$ to $200\ {\rm meV}$ [45,77,78], in line with the theoretical prediction. However, the photon-energy dependent gap size indicates its bulk nature rather than a surface origin. Indeed, our systematic photon-energy-dependent ARPES measurements show that the bulk gap separating the bulk valence and conduction bands varies from 130 meV to 200 meV from bulk Brillouin zone ${\mathrm{\Gamma }}$ to *Z* [50]. Astonishingly, the TSS Dirac cone remains gapless below or above ${T}_N\sim 24.6\ {\rm K}$, as first reported by our group (data shown in Fig. 2a) and others [50–52]. Such observations of (nearly) gapless TSS Dirac cone on MnBi_2_Te_4_ (0001) surface below and above the antiferromagnetic order temperature have been further repeated [79–84]. To date, there have been extensive efforts to explain the origin of this gapless behavior, which will be reviewed in the following sections.

This striking violation against the theoretical picture has inspired intensive ARPES measurements extended to the antiferromagnetic heterostructure members of this family, i.e. MnBi_4_Te_7_ consisting of alternating SL and QL sequence and MnBi_6_Te_10_ consisting of alternating SL and two QLs sequence [47,86]. The enlarged distance between SLs in MnBi_4_Te_7_ and MnBi_6_Te_10_ reduces the antiferromagnetic exchange interaction. Consequently, MnBi_4_Te_7_ has an antiferromagnetic ground state with ${T}_N\sim 13\ {\rm K}$, while MnBi_6_Te_10_ is antiferromagnetic below ${T}_N\sim 10.7\ {\rm K}$. [87–90]. Due to the vdW interaction between SLs and QLs, MnBi_4_Te_7_ has two natural cleavage planes (SL and QL terminations) while MnBi_6_Te_10_ has three (SL, QL, and double QL terminations). Since the intralayer ferromagnetic order comes from Mn residing in the central layer of SL, one would expect magnetic gap opening from SL termination and gapless TSS from QL and double QL terminations. However, similar to the case of MnBi_2_Te_4_ (0001) surface, all the SL terminations from MnBi_4_Te_7_ [52,79,85,91] and MnBi_6_Te_10_ [79,89,90,92] show (nearly) gapless TSS Dirac cone as presented in Fig. 2b and c. These results suggest that the (nearly) gapless behavior of the TSS Dirac cone is ubiquitous for all the SL terminations of (MnBi_2_Te_4_) · (Bi_2_Te_3_)_n_ ($n\ = \ 0,\ 1,\ 2$).

It is also interesting to look at the TSS behavior from QL and double QL terminations. Both QL terminations from MnBi_4_Te_7_ (Fig. 2b, right) and MnBi_6_Te_10_ (Fig. 2c, middle) present similar features for the TSS. First of all, the TSS Dirac point is buried inside the bulk valence band region as indicated by blue arrows. Second, an apparent gap is opened at the upper TSS Dirac cone due to its hybridization with one neighboring bulk valence band. Third, below this hybridization gap, the residual TSS and bulk valance band compose a Rashba-split band (RSB) feature, with the new RSB Dirac point coming from the original TSS Dirac point. Last and more importantly, there appears a new band inside the hybridization gap, with its top touching the upper part of the gapped TSS, forming a new gapless Dirac cone. This in-gap state extends from the new Dirac point down to the valence band region, resulting in the generally gapless surface and bulk spectra. The appearance of this new in-gap Dirac cone is intriguing. Based on the above band features, a TSS-RSB hybridization picture has been proposed to explain the complicated band features from both SL and QL terminations [90]. Combining circular dichroism ARPES and first-principles calculations, the existence of RSB and its hybridization with TSS have been firmly established by works from several groups [77,83,85,90,93]. The TSS-RSB hybridization can be simulated in a tight-binding simulation. By tuning the hybridization strength, the QL ARPES spectra can be reproduced. According to the simulation, the new in-gap Dirac cone indeed comes from the original bulk RSB (see Fig. 4b–d in ref. [90]). This hybridization picture can also reproduce well the ARPES spectra on the SL termination and potentially explain the puzzling gapless behavior of the TSS Dirac point, which will be discussed in detail in the following section. For the double QL termination of MnBi_6_Te_10_, similar band hybridization features are observed (Fig. 2c, right), but the hybridization gap is too narrow for the investigation of the in-gap state.

## KEY PROPERTIES RELATED TO THE TSS GAP

Since its first observation, attempts had been made to explain the gapless behavior at the Dirac point of the SL termination with *S* breaking under antiferromagnetic order [50–52]. Before going to the bewildering variety of proposals, we would like to mention the key properties established by various experimental probes, which are closely related to the gapless/gapped behavior of TSS at the SL.

**Figure 3.**
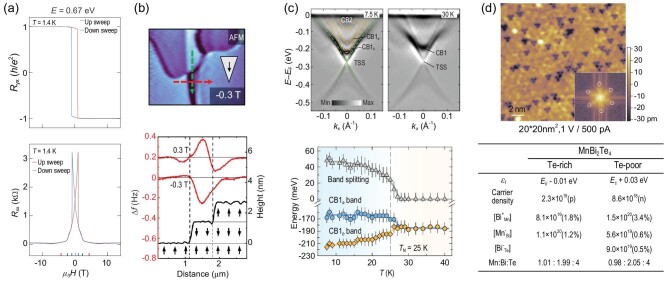
Key physical properties related to the gap behavior of TSS in Mn-Bi-Te family. (a) Realization of QAHE reveals an extremely small TSS gap ($\Delta E\ = \ 0.67\ {\rm eV}$), from [63]. (b) Robust uniaxial A-type AFM order to the surface layers and antiferromagnetic domains observed using MFM, from [96]. (c) Bulk conduction band splitting related to the antiferromagnetic order as observed directly in ARPES spectra, from [51]. (d) Top panel shows two types of surface point defects (bright dots and dark triangles, probably corresponding to Bi_Te_/Mn_Te_ and Mn_Bi_, respectively) found in atomically resolved topographic image, from [49]; Bottom panel lists the calculated Fermi level (${\varepsilon }_f$), free carrier density (and the type of the carrier), as well as densities of the most important intrinsic defects (and concentrations in atomic %) at both Te-rich and Te-poor limits in MnBi_2_Te_4_, from [97].

The first one comes from the experimental realization of QAHE in a 5 SLs MnBi_2_Te_4_ device [63], as shown in Fig. 3a, strongly suggesting the existence of a 2D Chern insulator state with gapped TSS. By fitting the Arrhenius plot of longitudinal resistance ${R}_{xx}$ as a function of $1/T$, the energy gap of the thermally activated charge transport can be obtained as $\Delta E\ = \ 0.64\ {\rm meV}$ at zero-field [63]. This energy scale characterizes the minimum energy required to excite an electron from the surface valence band to the surface conduction band, two orders of magnitude smaller than the predicted exchange gap [45]. On the other hand, the gapless behavior of the TSS observed by ARPES may just represent the resolving power of the instrument, and the TSS gap could still exist but be smaller than the energy resolution (typically $> 1\ {\rm meV}$). The TSS gap size from ARPES measurements varies from being diminished [50,51], to $\sim 10\ {\rm meV}$ [52] or even larger, with strong sample and spatial dependence [94,95]. The above observations suggest that the gapless behavior of the TSS Dirac point at SL termination at the antiferromagnetic phase is unlikely to be symmetry enforced. It is worth noting that, according to the theoretical definition of antiferromagnetic TI, the TSS is protected in a weaker sense than the 3D strong TI, indicating that it is generally not stable to disorder [38].

The second key property is the robust A-type AFM order at the surface SL layers as evidenced by measurements using MFM [96] and other techniques. Fig. 3b shows the MFM image taken after field cooling at $0.6\ {\rm T}$, with the tip polarized by a magnetic field of $- 0.3\ {\rm T}$ perpendicular to the sample surface. Clear contrast in the image illustrates several domains, where the magnetic signal changes its sign when crossing the domain walls. If the magnetic moment on the tip is reversed by a magnetic field of $0.3\ {\rm T}$, all the domains and domain walls change their contrast. Further analysis of the screening effect from fractional QL impurity phases supports the persistence of uniaxial A-type spin order at the top SL layers. One may wonder if the robustness of A-AFM at the top SL layers is also sample dependent. It is thus important to note that other techniques, such as polar reflective magnetic circular dichroism (RMCD) [98] and X-ray magnetic circular/linear dichroism (XMCD/XMLD) [45,48,94,99–101] spectroscopies, also give strong evidence for the existence of net out-of-plane magnetic moments at the sample surfaces.

Even though the robust A-type AFM order is confirmed, what about its influence on the low-energy electronic structure? The (nearly) gapless behavior and its weak temperature dependence across the magnetic transition suggest a negligible effect of the magnetic order on the TSS. However, there are other bands close to the Fermi level which are surprisingly affected by the magnetic order. As exemplified in Fig. 3c by the bulk conduction bands labeled as CB1_a_ and CB1_b_, at high temperatures they merge into one band CB1, while their splitting starts when the temperature decreased to ${T}_N$ and reaches $\sim 35 - {\mathrm{to}}\ 45\ {\rm meV}$ below $10\ {\rm K}$ [51,52,102]. It is further reported that CB2 also shows a Rashba-like feature and band splitting below ${T}_N$ [80]. These results demonstrated the third key property of MnBi_2_Te_4_, i.e. the coupling between antiferromagnetic order and the low-energy bands.

The fourth one comes from a material point of view concerning the disorders typically present in transition metal chalcogenides. In MnBi_2_Te_4_ family, various types of disorders, such as ${\rm B{i}_{Te}}$ antisites (i.e. Bi atoms at the Te sites) located in the surface layer, ${\rm M{n}_{Bi}}$ substitutions (Mn-Bi intermixing) in the second and central atomic layer, and Mn vacancies (${V}_{Mn}$) are observed by combining many experimental tools such as scanning transmission electron microscopy (STEM), scanning tunneling microscopy (STM), single-crystal X-ray diffraction (XRD), energy dispersive X-ray (EDX) analysis in scanning electron microscope (SEM) [48,49,78,103–112] and even density functional theory (DFT) calculations [97]. We will show in the next section that certain types of disorders may strongly affect the magnetic response of the TSS.

## MAGNETIC RECONFIGURATION TO EXPLAIN THE (NEARLY) GAPLESS TSSs

**Figure 4.**
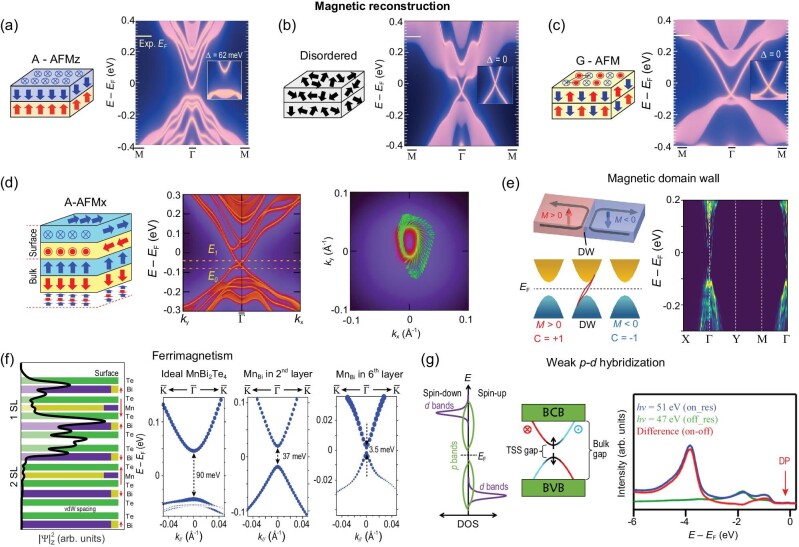
Various magnetic reconfigurations accounting for the (nearly) gapless behavior of MnBi_2_Te_4_ TSSs. (a–c) Prototypical magnetic reconstructions leading to different gap behavior of TSS, including A-type AFM with out-of-plane ferromagnetic moments (A-AFMz) (a), disordered magnetic moments (b), G-type AFM (c) (adapted from [50]) and A-type AFM with the magnetic moments along the *x*-axis (A-AFMx) (d) from [113]. (e) Magnetical domain wall edge states (left, from [28]) resemble the gapless TSS, which is further discussed in a first-principles-based tight-binding model (right, from [115]). (f) Native point defects ${\rm M{n}_{Bi}}$ can introduce ferrimagnetism and reduce the TSS gap, from [103]. (g) The hybridization between the Mn $3d$ and Te $5p$ states, as schematically shown in the left two panels (from [73]) is too weak according to the resonant photoemission spectra (right, from [52]) to induce TSS magnetic gap.

The (nearly) gapless behavior of TSS and its weak temperature dependence across the antiferromagnetic order lead to natural speculation of magnetic surface reconstruction. Deviations from the A-AFMz type (Fig. 4a), such as disordered magnetic structure (Fig. 4b), G-AFM with intralayer and interlayer antiferromagnetic (Fig. 4c) and AFMx with ferromagnetic in-plane moments (Fig. 4d), are considered in calculations [50,69,71,113]. As shown in Fig. 4b–d, all three deviations can lead to gapless TSS. Since it remains a technical challenge to determine the magnetic structure for the topmost SL, it would be insightful to examine the specific features from ARPES spectra and check their correspondence to the various magnetic structures. As shown in the right panel of Fig. 4d, for A-AFMx, the net magnetic moments break the in-plane rotation symmetry and lead to a TSS constant energy contour with only mirror symmetry. Further consideration of spin texture will result in mirror symmetry breaking [113]. For G-AFM, the double-sized unit cell in the antiferromagnetic phase may lead to in-plane band folding feature compared to the paramagnetic phase. For the disordered case, the lack of any oriented moment would retain the sixfold rotation symmetry of the constant energy contour of the TSS, while for the A-AFMz, the net out-of-plane moments in the top SL layer coupled to the TSS will break the sixfold rotation symmetry and leave only a threefold rotation symmetry. Further ARPES studies with ultrahigh energy, momentum resolution, and spin resolution from three directional components (${P}_x,\ {P}_y,\ {P}_z$) are highly encouraged to distinguish the above features.

Another type of magnetic reconfiguration is the formation of domains and domain walls illustrated in Fig. 4e (left panel [28]). The existence of magnetic domain walls in MnBi_2_Te_4_ surface has been observed experimentally [96,114]. In the presence of such opposing magnetic domains, first-principles calculations and tight-binding model analysis proposed that gapless chiral boundary modes can exist [37] (Fig. 4d, right panel [115]). Note that this edge mode is strictly gapless, while the TSS gap size in MnBi_2_Te_4_ shows sample and spatial dependence [94,95]. As the typical magnetic domain size is ∼10 μm, the contribution of chiral edge states at domain walls could be too tiny to explain the gapless topological surface states [96].

One more sophisticated ferrimagnetic structure has been experimentally observed [107,108] and employed to account for the much-reduced TSS gap [103]. As shown in Fig. 4f, the Mn-Bi intermixing could introduce ${\rm M{n}_{Bi}}$ defects in the 2nd and 6th atomic layers counting from the surface, with its moments antiparallel to that of the central Mn layer. Due to the predominant localization of TSS density of states to the Te-Bi-Te layer, the moments of ${\rm M{n}_{Bi}}$ defects counteract that from the central Mn layer, leading to a reduction of the TSS gap. The inhomogeneity of ${\rm M{n}_{Bi}}$ defects could explain the sample and spatial dependence of TSS gap size, suggesting that improving the crystalline quality with suppressed Mn-Bi intermixing is a crucial task for studies in the near future [103]. However, to better correlate the ${\rm M{n}_{Bi}}$ defect density to the TSS gap size, one needs to perform *in situ* ARPES and STM measurements for the same region of the sample surface, which is hardly feasible considering that these two techniques have ‘field of view’ with orders of magnitude difference.

It is also reasonable to check the effective coupling between Mn *d* orbitals contributing magnetism and Bi/Te *p* orbitals related to the TSS. According to a resonant photoemission study [52], the Mn $3d$ states are mainly located $4\ {\rm eV}$ below the Fermi level (Fig. 4g), negligible in the energy range where nontrivial topology arises, leading to the speculation of weak $p - d$ hybridization. This speculation is against first-principles calculations which predict sizable TSS gaps [116] and contradicts the observation of magnetism-induced conduction band splitting as discussed in ‘key properties related to the TSS gap’ (above). It is noted that a recent ultrafast magnetic dynamic study reveals large $p - d$ exchange coupling ($>\!\! 10\ {\rm meV}$) [117]. Based on the above analyses, there remain plenty of challenging experimental investigations, microscopically or spectroscopically, to determine the magnetic structure of the topmost SL accounting for the (nearly) gapless TSS.

## TSS REDISTRIBUTION TO EXPLAIN ITS (NEARLY) GAPLESS BEHAVIOR

In this section we introduce the TSS redistribution picture where the TSS distribution can be extended from the topmost SL to the layers beneath, leading to a (nearly) gapless TSS as it feels a compromised effective magnetic moment. The compensation of the effective magnetic moments relies on the fact that the topmost SL and the second SL have antiparallel and comparable moments, meaning that this TSS redistribution picture is only applicable to MnBi_2_Te_4_ but not the heterostructure members containing nonmagnetic QLs. However, the potential origins of a redistributed TSS, such as band hybridization, vdW spacing expansion, or charge/defect effect, may generally exist in all the members of this family. In the following, we briefly introduce these mechanisms.

**Figure 5.**
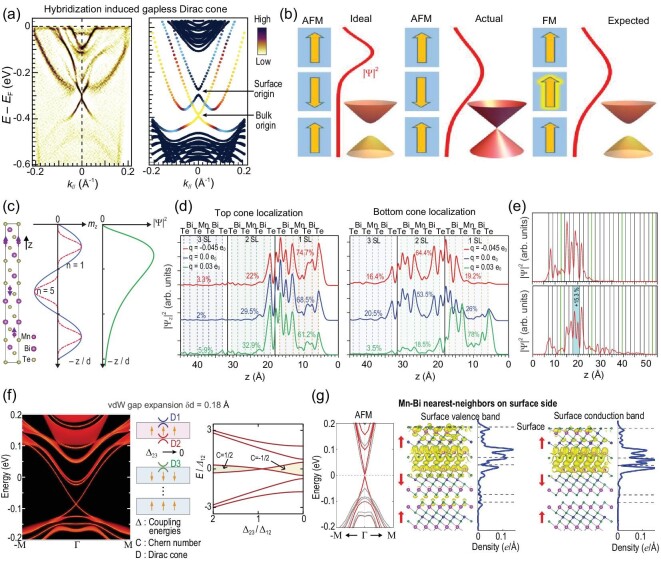
TSS redistribution picture explaining the (nearly) gapless behavior of MnBi_2_Te_4_ TSSs. (a) ARPES spectra and tight-binding model simulation show the hybridization between TSS and Rashba-split bands, from [90]. (b) Schematic showing the redistribution of TSS density of states and gapped/gapless Dirac cone corresponding to an ideal antiferromagnetic phase (left), an actual antiferromagnetic phase (middle) and an expected ferromagnetic phase (right). (c) Distribution of bulk magnetization (${m}_z$) and surface state envelope function ${\mathrm{\Psi }}$ according to effective model analysis, from [118]. (d) Surface excess charge induced redistribution of TSS top/bottom cone localization, from [95]. (e) TSS redistribution due to the vdW spacing expansion, from [94]. (f) Three-Dirac-fermion approach to the gapless TSS under vdW spacing expansion, from [119]. (g) Co-antisites (exchanging Mn and Bi atoms in the surface layer) can strongly suppress the magnetic gap down to several ${\rm meV}$ in the antiferromagnetic phase, from [120].

Based on the observation of hybridization between the TSS and a pair of RSBs, A TSS-RSB hybridization picture has been proposed [90] to explain the origin of sophisticated band structure for both QL and SL terminations in this material family (see details in ‘ubiquitouly gapless TSSs’ above). Specifically for SL as shown in Fig. 5a, tight-binding model simulation reveals that the TSS Dirac cone has a bulk origin. This inspires a TSS redistribution picture to account for the lack of magnetic effect on TSS in MnBi_2_Te_4_. As schematically illustrated in Fig. 5b, in an *ideal* case, the TSS predominantly locates on the topmost SL. In the A-AFM configuration, the effective magnetic moments for the TSS are approximately equal to the net ferromagnetic moments from one SL, which is large enough to open a sizable TSS Dirac gap as expected. In the *actual* case, the TSS distribution extends to the second SL. The interlayer antiferromagnetic order results in zero net magnetization for the top two SLs and consequently compensated effective magnetic moments for the TSS. In an extreme situation where the top two SLs equally share 50% of TSS localization, gapless TSS appears regardless of the robust surface A-AFM order and its coupling to the band structure. Based on this TSS redistribution picture, a sizeable TSS magnetic gap can be expected if the magnetic compensation effect is eliminated, e.g. in a ferromagnetic ground state. This *expected* case will be discussed in the next section.

Such a TSS redistribution picture has been supported by numerous model analyses and distinct calculations-based approaches. Starting from a 3D Hamiltonian for bulk MnBi_2_Te_4_ and taking into account the spatial profile of the bulk magnetization, an effective model for the TSS has been derived [118]. This model suggests that the diminished surface gap may be caused by a much smaller and more localized intralayer ferromagnetic order and the fact that the surface states are mainly embedded in the first two SLs from the terminating surface (Fig. 5c). To be specific, by using the envelope function the penetration depth of TSS is calculated as ∼16.2 $\mathring{\rm A}$, larger than the thickness of one SL (∼13.7 $\mathring{\rm A},$). This is in agreement with the results obtained by *ab initio* calculations, which present the spatial charge distribution of the TSS Dirac state between the first and second SL expanded by 15.3% at equilibrium structure and for vdW spacing (Fig. 5e from ref. [94]). With increasing vdW spacing, the TSS is found to shift its dominant occupation from the top SL to the second SL, resulting in reduced effective moments. The TSS Dirac gap is found to decrease and vanish at 15.3% expansion. As the vdW spacing modulation is likely to occur in both magnetic and nonmagnetic vdW TIs, a general three-Dirac-fermion approach can be developed [119] to describe the TSS behavior. As shown in Fig. 5f, the three-Dirac-fermion refers to three TSS Dirac cones located at the top surface of the topmost SL/QL (${D}_1$), the bottom surface of the topmost SL/QL (${D}_2$) and the top surface of the second SL/QL (${D}_3$), respectively. Their coupling is tuned by coupling energies ${{\mathrm{\Delta }}}_{12}$ and ${{\mathrm{\Delta }}}_{23}$, with the latter being dependent on the topmost interlayer vdW spacing *d*. Remarkably, unexpected gapless TSS Dirac cones are found to arise due to *d* expansion, when the total Chern number of the system changes by 1 in this expansion process. It should be emphasized that, in this three-Dirac-fermion approach, the gapless point is topologically protected and comes from the competition between the Zeeman coupling and the Dirac fermion coupling. Such vdW spacing expansion may be introduced by the mechanical cleavage process [22,121] before ARPES and STM measurements, yet the direct evidence is still missing from atomic layer resolved probes such as STEM.

Excess surface charge is found to affect the distribution of TSS in a top/bottom cone-dependent manner [95]. Obviously, the smallest gap values should be achieved when the top and bottom TSS cones are mostly and independently located in two adjacent SLs, as they experience an exchange field of the opposite sign ($q\ = \ - 0.045{e}_0$ in Fig. 5d). Furthermore, from an *ab initio* calculation, cation co-antisites ${\rm M{n}_{Bi}}$ and ${\rm B{i}_{Mn}}$ (extra Bi replacing Mn) can push the TSS charge toward the second SL. Therefore, the influence on the magnetic gap from the top SL is reduced while the second SL influence is simultaneously enhanced (Fig. 5g) [120], resulting in compensated effective magnetic moments and reduced TSS magnetic gap. It is noted that the existence of excess charge and Mn-Bi intermixing defects are well established in this material family.

## PERSPECTIVES TO OPEN THE TSS MAGNETIC GAP

In the context of the TSS redistribution picture, gapless TSS comes from compensated magnetic moments as a nature of the A-AFM order. Assuming a ferromagnetic background, no compensation exists no matter how the TSS redistributes. This offers a great chance to realize a sizeable TSS gap through magnetic engineering based on MnBi_2_Te_4_. In fact, a large amount of Sb substitution in the Bi sites can indeed transform the MnBi_2_Te_4_ ground state from antiferromagnetic to ferromagnetic or ferrimagnetic order [104,107,123–125]. The TSS band structure study by ARPES, however, is difficult due to the heavy hole doping induced by Sb substitution. It is worth noting that with a small amount of Sb doping, we have observed a TSS gap opening in MnBi_2_Te_4_ samples which stay at the antiferromagnetic phase. Surprisingly this TSS gap size is proportional to the doping level and carrier density, allowing a continuous tunability of gap size [126]. However, this TSS gap is independent of the antiferromagnetic-paramagnetic transition, with the origin of the gap remaining to be investigated.

**Figure 6.**
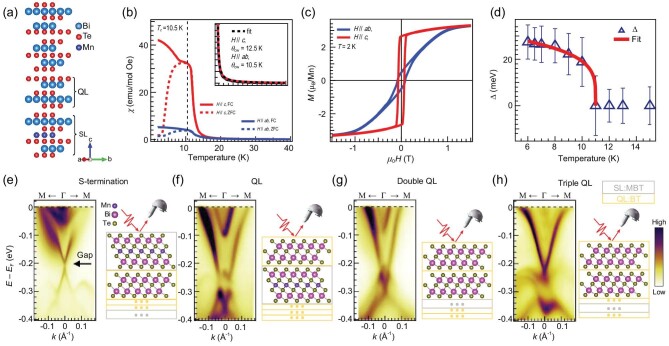
Realization of TSS magnetic gap from the S-termination of ferromagnetic MnBi_8_Te_13_, from [122]. (a) Schematic crystal structure with one unit of -SL-QL-QL-QL- sequences. (b) magnetic susceptibility shows the ferromagnetic order with Curie temperature ${T}_C = 10.5\ {\rm K}$. (c) Field-dependent magnetization hysteresis at $2\ {\rm K}$. (d) TSS gap size *vs* temperature shows its ferromagnetic origin. The gap size is extracted from the TSS of S-termination as shown in (e). (e–h) Termination-dependent band structure measured at $7\ {\rm K}$ in ferromagnetic state. The TSS Dirac gap is indicated by a black arrow in (e).

Another way to realize the ferromagnetic ground state is through heterostructure engineering. As mentioned in ‘ubiquitouly gapless TSSs’, in (MnBi_2_Te_4_) · (Bi_2_Te_3_)_n_ ($n = 0,\ 1,\ 2,\ 3$) family the interlayer antiferromagnetic coupling between the ferromagnetic SLs can be reduced by QL spacing. In fact, with 3 QLs spacing ($n = 3$), MnBi_8_Te_13_ develops a long-range ferromagnetic order below ${T}_C = 10.5\ {\rm K}$ [127]. This provides a valuable chance to realize the magnetic gap in TSS from SL termination. High-quality MnBi_8_Te_13_ single crystals are grown and characterized through structural, magnetic, transport, and electronic structure studies [122]. Its crystal structure shown in Fig. 6a was obtained from single-crystal XRD and powder XRD refinement. The temperature-dependent anisotropic magnetic susceptibility (Fig. 6b) shows Curie-Weiss (CW) behavior above $150\ {\rm K}$ (inset) with the characteristic temperature ${\theta }_{CW} = 12.5\ {\rm K}$ and $10.5\ {\rm K}$ for $H\parallel c$ and $H\parallel ab$, respectively. The larger bifurcation between zero-field cooling (ZFC) and field cooling (FC) magnetization and magnetic hysteresis loop (Fig. 6c) indicate an easy axis along the *c*-axis and an Ising-type exchange interaction between adjacent Mn layers. These properties suggest a ferromagnetic order with an out-of-plane magnetic moment configuration in MnBi_8_Te_13_.

The heterostructure lattice of MnBi_8_Te_13_ naturally yields four types of terminations by cleaving the single crystal perpendicular to the *c* axis, namely, SL, QL, double QL, and triple QL terminations. A spatial-resolved ARPES with a laser beam spot ∼$5\ \mu m$ was employed to resolve the intrinsic surface band structure from these four distinct terminations and the results are shown here in Fig. 6e–h. We start with those three nonmagnetic QL terminations and find very similar TSS-RSB hybridization features as we discovered from QL and double QL terminations of MnBi_6_Te_10_ (Fig. 2c [90]), while the triple QL termination shows a TSS Dirac cone resembling that of Bi_2_Te_3_. Emphasis was put on the SL termination and a clear gap can be found at the TSS Dirac point as indicated by the black arrow in Fig. 6e. The gap size is extracted by fitting the energy distribution curves (EDCs) using multiple Lorentzian peaks. The fitting yields a TSS Dirac gap $\sim 28\ {\rm meV}$ at $7\ {\rm K}$. Furthermore, this gap exhibits a monotonical decrease with increasing temperature (Fig. 6d) and finally closes at $11\ {\rm K}$, right above ${T}_C$, establishing a ferromagnetic-induced Dirac-point gap in the SL termination. Although TSS gaps have been observed in other members of this materials family and doped TIs, their magnetic origin remains controversial with, particularly, the lack of clear temperature dependence [50,51,79,90,128]. Consequently, this observation—that a TSS Dirac cone gap decreases monotonically with increasing temperature and closes right at ${T}_C$, forming a gapless Dirac cone—represents direct evidence of TSSs gapped by the magnetic order among all known magnetic topological materials. It is still more desirable to realize the magnetic gap of TSSs in MnBi_2_Te_4_ rather than its heterostructure cousins as the latter contains uncontrollable terminations with different magnetism from the exfoliation process. The realization of the TSS magnetic gap in ferromagnetic MnBi_8_Te_13_ seems to be consistent with the TSS redistribution picture.

We have discussed several possible microscopic mechanisms of TSS in two aspects, i.e. magnetic configuration (such as disordered magnetic structure and A-AFMx with ferromagnetic in-plane moments) and TSS redistribution (such as TSS-RSB hybridization, vdW spacing expansion, excess surface charge, and cation co-antistites effect). It should be noted that, to reach any of these situations, an energy barrier needs to be overcome due to its deviation from the bulk ground states. Hence, a natural question arises: why would these situations occur? Is it an occasional case for Mn-Bi-Te family or does there exist some general fundamental mechanism? From the perspective of energy competition, a more general self-doping scenario in real samples had been proposed as the essential force that may lead to such situations based on Koopmans’ theorem [129]. For the gapped TSS, the energy level of the conduction band minimum is higher than that for the case of the gapless Dirac point. According to Koopmans’ theorem, an electronic self-doping would naturally save energy for the gapless TSS. Once such energy gain overwhelms the energy barrier of the redistribution of the TSS or magnetization, the gapless behavior of TSS emerges. In this sense, the self-doping may be the underlying origin of the nearly gapless TSS, while the TSS redistribution or magnetic reconfiguration serves as the intermediate. In this view, the emergence of the magnetic gap of MnBi_8_Te_13_ could also be understood. Instead of staying at the surface SL that contributes to the TSS, most of the self-doped electrons enter the bulk QL bands, thus suppressing the gapless transition of TSS [129]. Therefore, to open the TSS magnetic gap it is favorable to recover its charge neutrality via doping or material optimizing.

Despite the remaining puzzles of the microscopic mechanism of the TSS, there are currently experimental advances that may be informative. Related to the vdW gap mechanism, point contact tunneling spectroscopy on MnBi_2_Te_4_ reveals the signature of the TSS gap, which indicates that a moderate pressure on the surface may deduce the vdW gap expansion to restore the effective magnetic moments for a gapped TSS [130]. Related to the excess surface charge and antisite effect, improving the crystalline quality is a direct way to eliminate such effects. Recent efforts in growing MnBi_2_Te_4_ single crystals using chemical-vapor-transport (CVT) methods [131,132] have reported samples marked with higher Mn occupancy on the Mn site, slightly higher ${\rm M{n}_{Bi}}$ antisites, smaller carrier concentration, and a Fermi level closer to the Dirac point, yielding highest mobility of $2500\ {\rm c{m}}^2{\rm V}^{ - 1}{\rm s}^{ - 1}$ in MnBi_2_Te_4_ devices [131]. ARPES measurement with ultrahigh energy and momentum resolution, as in the previous studies [50–52,122,126], is called for the exploration of spectroscopic signature of coupling between the ferromagnetic order and TSS, such as magnetic gap opening and sixfold rotation symmetry breaking [119,133]. Quantum transport measurement is highly expected for thin layer devices based on CVT single crystals in pursuit of quantized conductivity at higher temperature.
